# The Diagnostic Role of FDG PET/CT in Patients with Fever of Unknown Origin

**DOI:** 10.4274/MIRT.20.04

**Published:** 2011-04-01

**Authors:** Nurhan Ergül, Metin Halac, Tevfik F. Cermik, Resat Ozaras, Sait Sager, Çetin Onsel, Ilhami Uslu

**Affiliations:** 1 Istanbul University Cerrahpasa Faculty of Medicine, Department of Nuclear Medicine, İstanbul, Turkey; 2 Istanbul Education and Research Hospital, Clinic of Nuclear Medicine, Istanbul, Turkey; 3 Istanbul University Cerrahpasa Faculty of Medicine, Department of Infectious Diseases, İstanbul, Turkey

**Keywords:** Fluorodeoxyglucose F18; positron-emission tomography; fever of unknown origin; diagnosis

## Abstract

**Objective:** Fever of unknown origin (FUO) is a challenge for the physician and needs use of clinical, laboratory, and imaging studies and also invasive and/or non-invasive interventions to detect the etiology. The aim of present study was to assess the role of FDG PET/CT in determining the etiology in patients with FUO.

**Material and Methods:** Twenty-four patients (median age 52, range 5-77 years, 6 female, 18 male) who were diagnosed with FUO were retrospectively analyzed in this study. Before the FDG PET/CT studies, none of them had a definitive reason for their diseases investigated by conventional radiological or scintigraphic methods, clinical and laboratory observations.

**Results:** The positive result was achieved in 19 (79.2%) of 24 patients as findings of the FDG PET/CT. However, FDG PET/CT was useful for definitive diagnosis in 12 (63.2%) of 19 positive patients. Malignant diseases were determined to be the underlying cause of FUO in 5 (41.6%) of 12 patients. Noninfectious inflammatory causes were detected in 2 (16.7%) patients, infections were exhibited in 3 (25%) patients, and miscellaneous diseases demonstrated in 2 (16.7%) patients. In 7 patients the detected pathological uptakes on FDG PET/CT were not helpful for the definitive diagnosis. In remaining 5 patients who showed no pathological uptake in the FDG PET/CT, diagnosis could not be established by other methods, as well. The sensitivity, specificity, and positive and negative predictive values for the determination of FUO etiology were 92.3%, 45.4%, 63.1%, and 100% for FDG PET/CT.

**Conclusion:** Our results demonstrate that FDG PET/CT seems to have considerable contribution to reveal the reason of undiagnosed patients with FUO investigated by conventional diagnostic methods, clinical and laboratory observations.

**Conflict of interest:**None declared.

## INTRODUCTION

The classical definition of fever of unknown origin (FUO) was made by Petersdorf and Beeson in 1961 as “a fever that is measured to be above 38.3 0C on several occasions during a period longer than 3 weeks for which the etiology behind cannot be diagnosed at the end of at least 1 week hospital stay” (1). In 1991, Durack and Street have made two major changes on this definition which identified and separated FUO types (nosocomial FUO, neutropenic FUO, HIV-associated FUO) that require entirely different clinical approaches in diagnosis and treatment compared with the classical one; moreover, the requirement of at least 1 week hospital stay has been replaced with 3 days hospital stay or 3 outpatient visits (2). The prevalence of FUO among hospitalized adult patients is reported to be 2.9% (3). The spectrum of FUO etiology may include more than 200 diseases (2). The diseases causing FUO vary depending on geographical differences, development level of countries, and experience of the clinician (4). According to the studies conducted to date, the diseases taking part in FUO etiology and their rates are as follows: infections (21-54%), noninfectious inflammatory causes (13-24%), neoplasms (6-31%), and other causes (4-6.5%) (4). The rate of failure to reach a definitive diagnosis in patients with FUO, varies between 7-53% (5). 

In patients presenting with FUO, basic diagnostic methods are performed following detailed history and physical examination. As those methods can differ between clinics, generally the followings are employed: routine biochemical blood tests, complete blood count (CBC), peripheral blood film, urinalysis, blood cultures, and chest x-ray (2). In some centers, abdominal USG and CT along with tuberculin skin test are applied, as well (5). 

FDG PET is a valuable method for its success in demonstrating both neoplasms, and infection-inflammation foci. Currently, the contribution of FDG PET or PET/CT in FUO diagnosis has been reported to be 16-69% (6). The aim of the present study was to assess the diagnostic role of FDG PET/CT in determining the etiology in patients with FUO. 

## MATERIALS AND METHODS

**Patients**

In the present study, 28 patients who have been diagnosed as FUO and referred to investigation of the etiology of the fever were retrospectively analyzed by FDG PET/CT. Because adequate clinical information could not be reached in 4 of those patients, they were excluded from the study. The remaining 24 patients (6 female and 18 male, median age: 52 years [range 5-77 years]) were included in the study. 

All the patients complied with the FUO criteria (a continuous or repeated fever that is measured to be above 38.3 0C during a period longer than at least 3 weeks for which the etiology behind could not be diagnosed within 1 week of clinical investigation). None of the patients had an underlying disease that could lead to immunodeficiency. Median duration of patients’ fever before PET/CT scan was 75 days (range 22 days-1 year). All the patients had received basic diagnostic assessments including routine biochemical tests, CBC, peripheral blood film, urinalysis, blood cultures, and chest x-ray. Thoracic CT and abdominal CT had been performed in 80% and 65% of patients, and other diagnostic methods such as abdomen MRI and scintigraphic methods had been conducted in 21% and 8% of patients, respectively. In our study group, above mentioned conventional imaging methods and noninvasive laboratory tests were performed before FDG PET/CT scans, and there was not any definitive diagnosis of FUO in our patients. 

**FDG PET/CT Imaging**

All the PET/CT scans were performed by a high-resolution PET scanner with an integrated six-slice multidetector CT (Siemens Biograph LSO HI-RES PET/CT, Illinois, USA). Prior to FDG injection, blood sugar was measured in well hydrated patients who fasted at least 4 hours before their appointment. 296-703 MBq FDG was administered intravenously to the patients with a blood sugar level below 150 mg/dL. Following injection, patients were left to rest in a peaceful and comfortable room for 60 minutes in order to let FDG to complete its biodistribution throughout the body. At the end of this waiting period, bladders of the patients were emptied and they were instructed to lie down at supine position on the PET/CT scanner bed. Following noncontrast low-dose CT scan, vertex-to-toe whole body PET scan was performed. PET scans were completed with acquirement of 7-8 bed positions with 3-4 minutes of acquisition time per position from vertex-to-upper thigh. In lower extremities, PET scan was carried out with acquirement of 6-7 bed positions with 2-3 minutes of acquisition time per position from upper thigh-to-feet. 

**Image Analysis**

PET/CT images were visually and semiquantitatively assessed by an experienced nuclear medicine physician. The reconstructed images were visually assessed in the standard axial, coronal, and sagittal views. The accumulation of FDG outside the physiological uptake areas was considered pathological. For semiquantitative analysis, a region of interest was carefully drawn around the site of increased FDG uptake on the subsequent 4–10 PET scan slices and the maximum standardized uptake values (SUVmax) of the lesions were calculated and used for this analysis. The SUV was calculated using the following formula: SUV=Tissue concentration (Bq/g)/[Injected dose (Bq)/Body weight (g)].

The prognosis of the disease was followed-up for 3 months after the PET/CT study by conventional diagnostic examinations and procedures. Diagnosis of patients with detected pathologies was conducted by localized detailed imaging methods, biopsies, and other invasive procedures or clinical observation aimed at the pathological findings, whereas secondary evaluations and clinical examinations were employed in other patients. 

FDG PET/CT was considered to help FUO diagnosis (true positive) in cases where the pathological foci shown by PET/CT displayed consistency with the definitive diagnosis of the patients. Scans which have shown foci that were not consistent with the diagnoses (false positive), and scans where PET/CT showed no pathological foci despite determination of a foci by other methods or during the clinical follow-up (false negative) were considered to be unhelpful for the diagnosis. In cases where PET/CT was negative and no pathological focus could be determined, the result was considered as true negative. 

**Statistical Analysis**

Conventional methods were used to generate descriptive statistics and sensitivity, specificity and positive and negative predictive values were calculated based on their standard definitions. 

## RESULTS

Data on demographic characteristics of the patients, duration of fever, imaging studies prior to PET/CT, nonphysiological uptakes detected with PET/CT, established definitive diagnosis, and contribution of PET/CT to diagnosis, are given in Table 1. 

**Figure 1:**
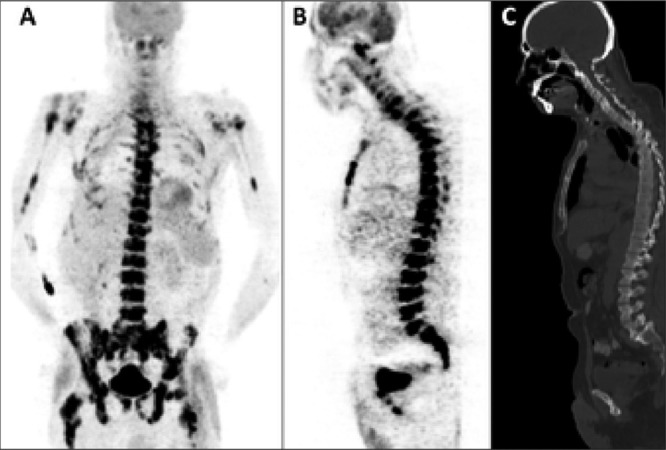
PET images of anterior MIP (A), sagittal PET (B) and CT(C) showinvolvement of bone marrow in several anatomical parts, the most prominentones being vertebral column, pelvic bones, and proximal femur. Bonemarrow biopsy revealed large B cell lymphoma

**Figure 2:**
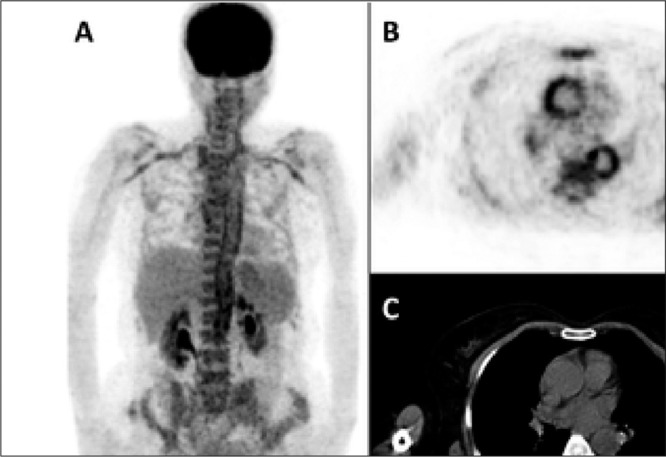
PET images of anterior MIP (A), transaxial PET (B) and CT (C)show involvement in large vessels (aorta, subclavian arteries, andcarotid arteries). A biopsy of temporal artery revealed giant cell arteritis(temporal arteritis)

**Figure 3:**
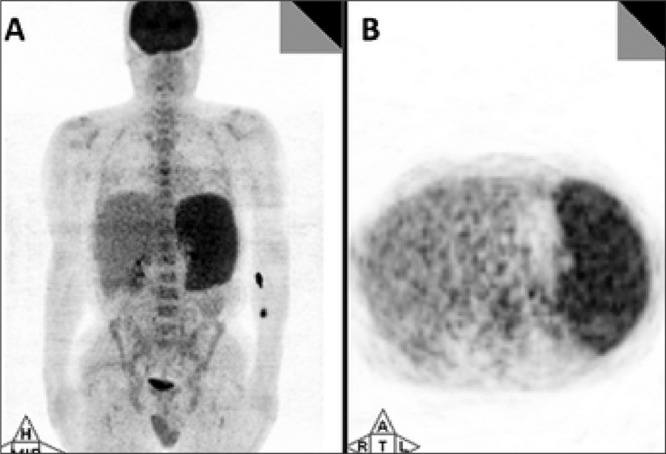
Anterior MIP (A) and transaxial (B) PET images showhepatosplenomegaly and a diffuse involvement of spleen. After PET/CT scanseveral new smears were prepared and diagnosed as having babesiosis

Pathological findings were detected by FDG PET/CT in 19 (79.2%) of 24 patients with FUO. The definitive diagnosis found to be consistent with the foci indicative of pathological uptakes determined by FDG PET/CT in 12 (63.2%) of 19 patients. Neoplasms were determined to be the underlying cause of FUO in 5 (41.6%) of 12 patients with definitive diagnosis (Figure1). In 2 (16.7%) patients displayed noninfectious inflammatory causes (Figure 2), 3 (25.0%) patients exhibited infections (Figure 3), and 2 (16.7%) patients demonstrated other diseases (Table 2). In 6 patients (pts 3,4,5,11,13,24) the detected pathological uptakes were not found useful for diagnosis and reason of fever could not be established by any other methods. One patient (pt 14) who was diagnosed with polymyalgia rheumatica by biopsy, demonstrated pathological uptakes in the liver but those findings were evaluated as hematomas by MRI and these 7 patients’ (36.8%) FDG PET/CT findings were considered as false positive.

In 5 patients (pts 2,8,16,19,20) who showed no pathological uptake in the FDG PET/CT, diagnosis could not be established by other diagnostic methods, as well. Definitive diagnosis was reached in 12 (50%) of 24 patients as a result of the FDG PET/CT findings.

Definitive diagnoses were confirmed via biopsy in 4 patients (pts 6,7,9,15), the verification was established by operation in 3 patients (pts 1,10,23). Because laparoscopic biopsy did not contribute to a patient (pt 22) who exhibited abdominal hypermetabolic lympadenopathies in FDG PET/CT, laparotomy was performed and the case was diagnosed as Castleman’s disease. In 5 patients, diagnosis was achieved through PET/CT findings, clinical observation, specific laboratory tests, and other imaging modalities (pts 12,14,17,18,21). 

In determination of FUO etiology, FDG PET/CT demonstrated 92.3% sensitivity, 45.4% specificity, 63.1% positive predictive value, and 100% negative predictive value, respectively. 

## DISCUSSION

Due to wide variety of etiology of FUO, physicians still experience difficulties in selecting and applying the diagnostic procedures in these cases. Because morphological alterations may not occur at early periods of infections and inflammatory processes, both of which constitute the bulk of the FUO etiology, the sensitivity of anatomical imaging modalities such as USG, CT, and MRI could be low. Moreover, since these modalities only show certain parts of the body, they cannot provide information on pathological events in systemic disorders (7). The rate of failure to reach a definitive diagnosis varies between 7-53% in the literature (5). Moreover, the rate of undiagnosed patients is reported to increase in the recent years (2,5). In cases where imaging modalities are not successful and fine needle aspiration biopsies or excisional biopsies fail, exploratory laparotomy may be performed. It can be of help particularly in tuberculosis and hematological malignancies (8). Tuberculosis infection constitutes a considerable portion of FUO etiology particularly in developing countries (9). 

FDG PET or PET/CT scan has been reported to be effective in the detection of malignancies as well as determination of extent of disease, treatment response and prognosis. Since the most common 3 etiologies of FUO are known to be infections, noninfectious inflammatory events and neoplasms, FDG PET scan appears to be a valuable modality in diagnosing the etiology of FUO.

In the current study, contribution of FDG PET/CT to establishment of a diagnosis in patients with FUO was 50%. Because our study group was selected from patients who had undergone conventional imaging and laboratory methods, 50% diagnostic ratio of FDG PET/CT could be quite reasonable. The most common FUO etiology is infections followed by neoplasms and noninfectious inflammatory events with varying rates (2,3,4,9). However, in the present study, neoplasms are found to be more common than infectious diseases. The underlying etiologies found in true positive FDG PET/CT scans were malignant diseases in 41.6%, infections in 25.0%, noninfectious inflammatory diseases 16.7%, and other reasons in 16.7%. This result is probably due to elimination of tuberculosis and brucellosis by applying specific diagnostic tests at the beginning. Similar to the previous studies in the literature, lymphomas have been found to be the most common malignant disease among patients with neoplasms in our study group (4,10). 

In previous studies which utilized FDG PET for diagnosis of FUO, contribution of FDG PET to the diagnosis has been reported to be between 16-69% (6,7,11,12,13,14,15,16,17). Furthermore, in the current study, the sensitivity, specificity, positive predictive value, and negative predictive value of FDG PET/CT were found to be 92.3%, 45.4%, 63.1%, and 100%, respectively. Relatively low results for specificity and positive predictive value are associated with the high number of false positive results (n=7). 

The study of Lorenzen et al (12) is one of the first studies using FDG PET in diagnosis of FUO and they found the contribution of FDG PET to establishment of diagnosis in a group of 16 patients, as 69%. They noted the absence of a pathological focus which could be the underlying cause for fever among patients with negative FDG PET results, and reported a high negative predictive value for FDG PET (12). In the present study, similarly, no diagnosis could be reached by other diagnostic methods in 5 patients who also did not demonstrate pathological uptake in the FDG PET/CT. 

Bleeker-Rovers and colleagues (13) retrospectively studied the contribution of FDG PET to the diagnostic process of patients in whom FUO or suspicious infection and inflammation foci were investigated. While diagnosis could be reached in 46% of 35 patients with FUO, they found the contribution of FDG PET to diagnosis, as 37%. They reported sensitivity, specificity, positive predictive value, and negative predictive value of FDG PET as 93%, 90%, 87%, 95%, respectively (13). Another study by the same group evaluated the place of FDG PET in FUO diagnosis prospectively. Diagnosis was reached in 50% of 70 patients and the contribution of FDG PET to the diagnosis was reported to be 33%. The sensitivity, specificity, positive predictive value, and negative predictive value of FDG PET was found 88%, 77%, 70%, and 92%, respectively. In the same study, abdominal and thoracic CTs were performed in a subgroup of 43 patients. The positive and negative predictive values of abdominal and thoracic CT were 48% and 86%, respectively (14). 

Buysschaert et al conducted a study on 74 patients with FUO and succeeded to diagnose 53 of them and found the contribution of FDG PET as 26% (15). Jaruskova et al reported contribution of FDG PET or PET/CT to the diagnosis as 36% among 118 patients (94 had FUO) with prolonged fever (7). Federici et al performed a study on 14 patients (10 with FUO and 4 with prolonged inflammatory syndrome) and reported the contribution of FDG PET to the diagnosis as 50% (6). 

There are also studies which compare the FDG PET and nuclear medicine methods in FUO diagnosis (11,16,17). Meller et al (11) performed a study and found the contribution of FDG PET to the diagnosis as 55% which was performed by a double-head coincidence camera in 20 patients with FUO. The sensitivity, specificity, positive and negative predictive values of FDG PET were found to be 81%, 86%, 92%, and 75%, respectively. The sensitivity, specificity, positive and negative predictive values of Ga 67 in this study were reported to be 67%, 78%, 75%, and 70%, respectively (11). Because of disadvantages of Ga 67 scan such as high radiation dose, long duration of procedure and evaluation, low spatial resolution, and low sensitivity, specificity, positive and negative predictive values, FDG PET is a more valuable method (11,18,19). 

Giant cell arteritis and Takayasu arteritis constitute 17% of all FUO causes. FDG PET has been reported to be superior to other imaging modalities in detection of vasculitis (11,6,16). FDG uptake has been shown in cases of giant cell arteritis, polymyalgia rheumatica, Takayasu arteritis, peritonitis associated with Wegener granulomatosis, and infectious vasculitis (20). The sensitivity and specificity of FDG PET in detection of vasculitis have been reported to be 77-100% and 89-100%, respectively (21). CT and MRI are employed for diagnosis of Takayasu arteritis, however, FDG PET has been found to be more effective especially for lesions of early stage (22). In the current study, vasculitis was diagnosed in all the 3 patients with noninfectious inflammatory causes. 

The design of the present study had some limitations. First, there was a lack of the reason of false positive results of FDG PET/CT in six patients in follow-up period. Second, we consider the small number of patients with FUO as a deficiency of this report. 

In conclusion, the results of the present study demonstrate that FDG PET/CT seems to have considerable contribution to reveal the reason of undiagnosed patients with FUO investigated by conventional diagnostic methods, clinical and laboratory tests. Therefore, routine use of FDG PET/CT in assessing FUO is well justified.

## Figures and Tables

**Table 1:**
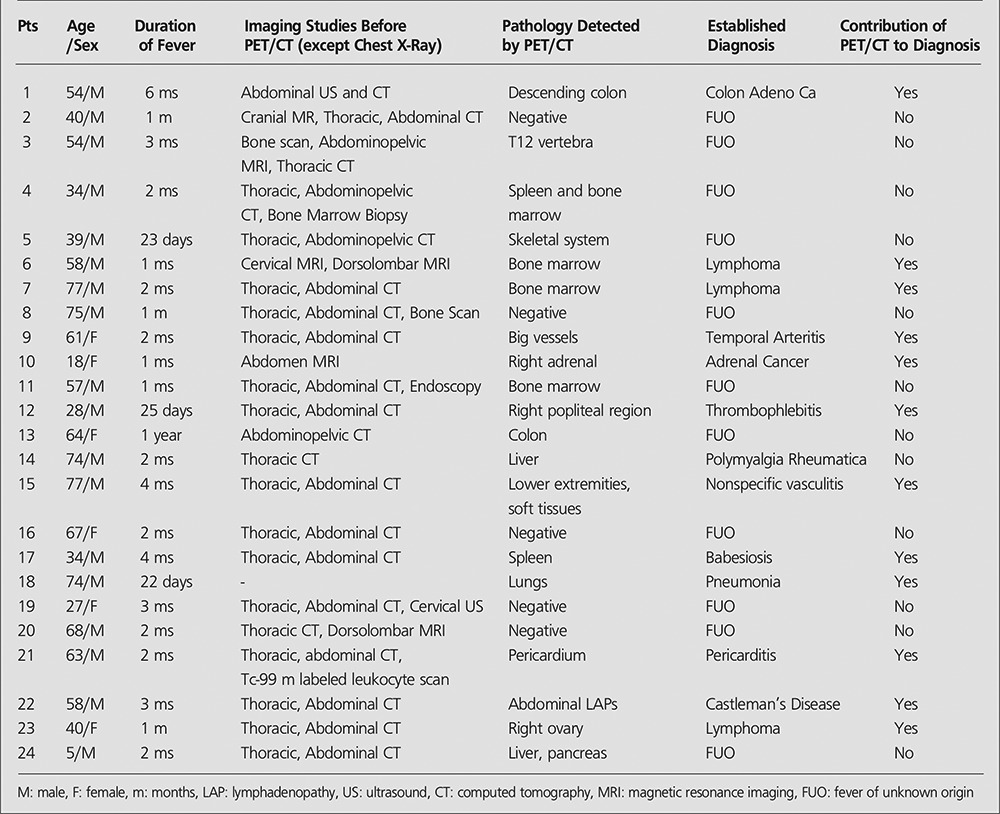
The patients’ characteristics, FDG-PET/CT results and final diagnosis

**Table 2:**
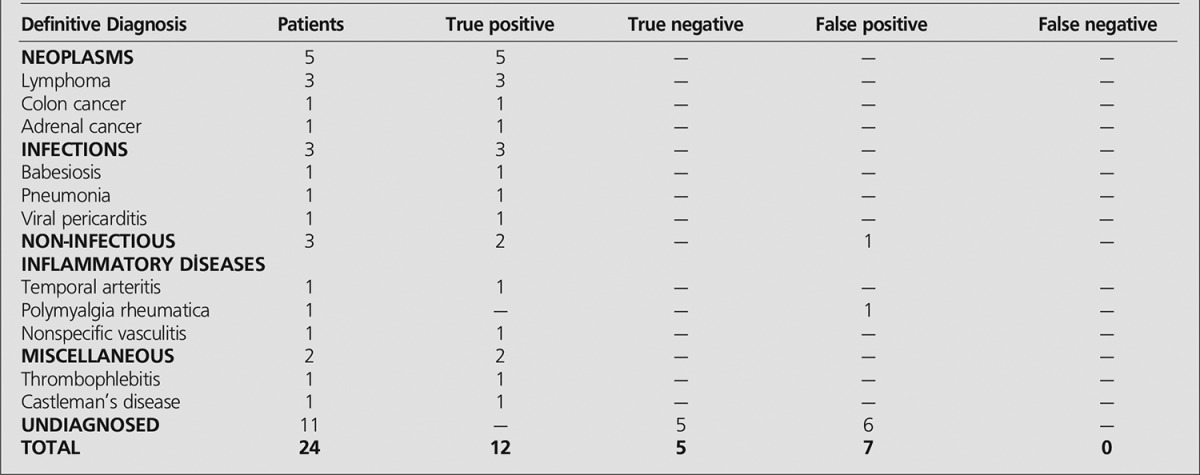
The definitive diagnosis of patients with fever of unknown origin
